# KG-SR-LLM: Knowledge-Guided Semantic Representation and Large Language Model Framework for Cross-Domain Bearing Fault Diagnosis

**DOI:** 10.3390/s25185758

**Published:** 2025-09-16

**Authors:** Chengyong Xiao, Xiaowei Liu, Aziguli Wulamu, Dezheng Zhang

**Affiliations:** 1 School of Automation & Electrical Engineering, University of Science and Technology Beijing, Beijing 100083, China; xcyustb@ustb.edu.cn; 2 School of Computer & Communication Engineering, University of Science and Technology Beijing, Beijing 100083, China; zdzchina@ustb.edu.cn

**Keywords:** bearing fault diagnosis, Large Language Model, knowledge-guided fine-tuning, domain knowledge fusion, cross-domain generalization

## Abstract

**Highlights:**

**What are the main findings?**

- Vibration signals can be transformed into structured and interpretable semantic sequences.
- The proposed KG-SR-LLM with LoRA-Prompt fine-tuning achieves robust cross-domain diagnostic performance.

**What is the implication of the main finding?**

- The method enables efficient adaptation of LLMs to industrial fault diagnosis.
- It provides a generalizable framework validated across 11 diverse datasets from multiple industries.

**Abstract:**

Bearing fault diagnosis is crucial for stable operation and safe manufacturing as industry intelligence becomes increasingly advanced. However, under complicated non-linear vibration modes and multiple operating conditions, most of the current diagnostic methods are limited in terms of cross-domain generalization. To address these issues, this study develops a generalized diagnostic framework leveraging Large Language Models (LLMs), integrating multiple enhancements to improve both accuracy and adaptability. Initially, a structured representation approach is designed to transform raw vibration time series into interpretable text sequences by extracting physically meaningful features in both time and frequency domains. This transformation bridges the gap between sequential sensor data and semantic understanding. Furthermore, to explicitly incorporate bearings’ structural parameters and operating condition information, a knowledge-guided prompt tuning strategy based on Low-Rank Adaptation (LoRA-Prompt) is introduced. This mechanism enables the model to adapt more effectively to varying fault scenarios by embedding expert prior knowledge directly into the learning process. Finally, a generalized fault diagnosis method named Knowledge-Guided Semantic Representation and Large Language Model (KG-SR-LLM) is established. Large-scale experiments using 11 public datasets from industrial, aerospace, and energy fields are carried out to extensively evaluate its performance. Based on experiment analysis and a comparison of results, KG-SR-LLM is superior to classical deep learning models by 9.22%, reaching an average diagnostic accuracy of 98.36%. KG-SR-LLM is effective for handling few-shot transfer and cross-condition adaptation tasks. All these results illustrate the theoretical significance and application benefit of KG-SR-LLM for intelligent fault diagnosis of bearings.

## 1. Introduction

In the industrial field, mineral processing equipment plays a critical role in the beneficiation process, with bearings serving as core components whose operational state directly influences the equipment’s overall performance and reliability [1,2,3]. During prolonged operation, especially under high load, strong impact, and harsh operating conditions, bearings are highly susceptible to factors such as load, temperature, and corrosion, which can lead to wear, fatigue, corrosion, and other fault problems [4]. These problems may result in unplanned downtime, product interruptions, or even severe safety incidents, causing significant economic losses for enterprises. Therefore, timely and accurate fault diagnosis of bearings holds substantial engineering significance and economic value [5].

Conventional fault diagnosis methods always depend on signal analysis and machine learning algorithms [6,7,8], which demonstrate efficacy in relatively uncomplicated scenarios but are ineffective when confronted with complex non-linear data and are unable to analyze large-scale data. Furthermore, these methods are generally customized for particular devices or datasets, lacking sufficient cross-domain generalization to face fault diagnosis across different equipment and operating conditions.

In recent years, deep learning has greatly advanced image recognition and natural language processing, also opening up new paths for fault diagnosis. In particular, the development of Large Language Models (LLMs) has provided powerful tools for time-series data tasks. However, it is still not an easy task to utilize LLMs for fault diagnosis of bearings. On the one hand, most LLMs are designed for text input, whereas bearing vibration signals are high-dimensional time series, and direct input of such data may hinder the model’s ability to capture important temporal information. On the other hand, distributions of bearing data tend to be greatly different from equipment to equipment and from operating condition to operating condition, which brings great challenges to effective knowledge transfer and cross-domain generalization.

In an effort to alleviate these issues, this paper presents an LLM-based fault diagnosis framework referred to as Knowledge-Guided Semantic Representation and Large Language Model (KG-SR-LLM). The framework improves the fault feature capture ability of the model by converting bearing vibration data to semantic text and directly introducing bearings’ structural parameters, as well as operation condition knowledge, into it. On several public datasets, comparisons confirm that KG-SR-LLM attains an average accuracy of 98.36%, much higher than baselines, and is also very adaptable in few-shot transfer settings.

The main contributions of this paper are summarized as follows:

**1. Sequence-to-Semantic Cross-Modal Representation of Vibration Signals**: A novel cross-modal representation approach is proposed to transform raw vibration time-series data into semantic text sequences. By leveraging multi-dimensional time–frequency domain feature abstraction, the proposed structured representation preserves the physical interpretability of vibration signals while enabling Large LLMs to process complex sensor data within a unified semantic space. This design bridges the gap between sequential physical measurements and semantic reasoning capability.

**2. Domain prompt-guided fine-tuning strategy**: A LoRA-Prompt fine-tuning method guided by domain knowledge is designed for LLMs, where bearings’ structural parameters and operating condition information are introduced explicitly by means of prompt engineering. The method enhances the model’s fault feature identification and greatly improves its cross-domain generalization and diagnostic accuracy performance.

**3. Design and validation of a generalizable diagnostic framework**: A novel prompt-based diagnosis framework, KG-SR-LLM, is proposed. The framework is extensively validated on 11 public datasets of different industrial, aerospace, and energy domains. The performances of KG-SR-LLM have been proven to be superior, achieving an average diagnostic accuracy of 98.36% and largely surpassing classical deep learning baselines. KG-SR-LLM has also proved to be well adaptable in few-shot scenarios and under various operating conditions, which verifies its real-world practicability and generalization ability.

The remainder of this paper is structured as follows: Section 2 surveys recent studies of bearing fault diagnosis and highlights the main issues that this paper is designed to resolve. Section 3 describes the KG-SR-LLM framework in detail, as well as its most critical modules. Section 4 offers detailed experimental comparisons to prove the merits of the proposed method. Finally, in Section 5, this paper is concluded, and directions for future studies are presented.

## 2. Related Work

### 2.1. Bearing Fault Diagnosis

Bearing fault diagnosis is defined as the analysis of vibration data of running bearings to determine whether or not there is a fault and, if it is, to further identify its type (e.g., wear, pitting, cracks), position (e.g., inner race, outer race, rolling element), and severity (e.g., slight, moderate, severe). Timely and accurate diagnosis is of great importance. It ensures equipment’s reliability, improves the efficiency of manufacturing, and reduces maintenance costs. Fault diagnosis is classified into three main categories: machine learning-based methods, deep learning-based methods, and transfer learning-based methods. The following describes an overview of each of these categories.

Machine learning-based fault diagnosis methods often require feature engineering, which is extracted manually. These features are then fed into a classifier for training and prediction. A data-driven hybrid method was proposed by Toma et al. [9]. They used motor current signals, used genetic algorithms to select significant features, and ultimately fused multiple machine learning classifiers to increase fault diagnosis accuracy and reliability. Wang et al. [10] combined digital twinning with machine learning classification to predict bearing faults in real time and make decisions about improved maintenance. And they updated dynamic models and generated fault data through online learning. Although these approaches provide a high interpretability, they rely on manually created features and do not effectively process complex data.

Deep learning-based fault diagnosis methods can automatically obtain meaningful features from raw input data, eliminating the need for manual feature engineering, and directly predict the health status of bearings. They can learn effective representations from large-scale datasets but need to be trained using large volumes of labeled data and face interpretability limitations. Zhang et al. [11] transformed the raw vibration signal into a two-dimensional image and used a Convolutional Neural Network (CNN) to extract image features. To enhance diagnostic results in few-shot scenarios, noise disturbances, and varying operation scenarios, Tang et al. [12] introduced a Trusted Multi-scale Quadratic Attention embedded CNN (TMQACNN) that combines quadratic neurons, attention mechanisms, and multi-scale learning approaches. Chen et al. [13] created a Variational Kernel CNN (VKCNN) that is an interpretable diagnosis framework. It integrates Amplitude Modulation and Frequency Modulation (AM-FM) component extraction and residual attention mechanisms to enhance the model’s stability and interpretability in noisy environments. With an aim to enhance fault diagnosis accuracy, Wang et al. [14] presented a deep learning framework that combines one-dimensional (1D) and two-dimensional (2D) feature extraction. The framework utilizes both 1D signal features and hidden spatial representations from 2D data.

Despite the great success of fault diagnosis methods based on deep learning, two significant issues remain. First, the distribution of test data is normally different from that of real operating scenarios, which results in decreased model performance under practical applications. Second, it is time-consuming and labor-intensive to obtain large-scale fault data and label them, which greatly decelerates diagnostic efficiency. To overcome the above problems, transfer learning is brought into fault diagnosis, enabling the integration of collectively learned rich knowledge from different but related tasks to improve diagnostic accuracy with limited labeled data. Hou et al. [15] proposed a transfer learning approach based on simulated data, where fault pulses are constructed using pre-evaluating fault eigenfrequencies and combined with multi-head attention mechanisms to solve insufficient labeled data in the target domain. Zheng et al. [16] designed an unsupervised transfer learning method by combining structurally optimized CNN and fast batch kernel paradigm maximization, which increases diagnostic performance under noisy environments and minimizes reliance on labeled samples. Dong et al. [17] proposed a transfer learning scheme based on deep feature decomposition and type-level alignment, which greatly enhances diagnostic accuracy under various noise levels using two-stage feature extraction and optimization strategies. Sun et al. [18] proposed a Universal Domain Adaptation with Noisy Samples (UDANS) method based on a multi-branch CNN and classifier divergence optimization to detect noisy source samples, discover private target classes, and align domain distributions, achieving promising results on wheelset bearing datasets in rail transportation. Although very encouraging results have been achieved by these existing transfer learning solutions, there are still several limitations. First, there are domain adaptation strategies within most approaches, the generalization capabilities of which would deteriorate when there is a significant distribution shift between source and target domains. Second, such methods are often designed for use on particular network architectures or task setups, which undermines their scalability and applicability in heterogeneous scenarios. Third, they cannot include human expert knowledge into the diagnostic process, which decreases their interpretability and the possibility of fault analysis.

### 2.2. Large Language Model

By definition, LLMs are highly trained on large-scale dataset models, typically tens to hundreds of billions of parameters in number. LLMs acquire rich feature representations and context knowledge from large corpora and have widespread applications in fields such as Natural Language Processing (NLP) and Computer Vision (CV). Once applied to particular downstream tasks, LLMs can effectively transfer pretraining-crafted knowledge into task-specific performances.

In the field of NLP, the successful application of LLMs is a technological revolution. Classical models such as Bidirectional Encoder Representations Transformers (BERT) and Generative Pretrained Transformer (GPT) are able to achieve phenomenal performance through various downstream tasks using large-scale pretraining of an unsupervised type and task-specific fine-tuning. These models utilize self-attention mechanisms and heavy parallel computation to extract contextualized information and obtain deep semantic representations of text content. BERT has accomplished exceptional performances on various NLP tasks, such as text classification, named entity recognition, and question-answering tasks. The GPT family, owing to its generative capability and autoregressive architecture, also made great breakthroughs. Such achievements not only facilitated the development of NLP but also provided an impetus to employing LLMs in other application fields.

With the advances of LLMs in NLP, an increasing number of works have investigated their application in industrial fields, especially fault diagnosis. Differing from text and image data, bearing vibration signals belong to time-series data that have strong temporal dependencies and complicated frequency domain features, which bring specific modeling difficulties. Nevertheless, LLMs still hold great potential in this field. Peng et al. [19] enhanced transferability under different operating conditions by constructing a unified representation of the vibration signal. However, it is dependent on fault-free reference data, which restricts its application in real scenarios. Qaid et al. [20] introduced an FD-LLM framework, which formulates fault diagnosis as a multi-class classification task and utilizes the language understanding capability of the Large Language Model (LLM) to achieve higher diagnostic accuracy. However, it lacks adaptability in cross-machine component transfer. Tao et al. [21] presented an LLM-based diagnosis framework that enhances transferability using LoRA and QLoRA fine-tuning, but it fails to solve few-shot transfer scenarios. The above works provide meaningful insights and prove that LLMs have great potential in fault diagnosis, which is also the foundation of this paper.

## 3. Methodology

### 3.1. Overall Structure

The fault diagnosis framework proposed in this paper takes advantage of the strong representation learning capabilities of LLMs to achieve accurate and robust fault diagnosis. The design of the framework is flexible and extensible, allowing it to be applied not just to various operating conditions and bearing datasets but also to other vibration-based diagnostic problems covering components such as gears, rotors, and other similar mechanical systems.

**Figure 1:**
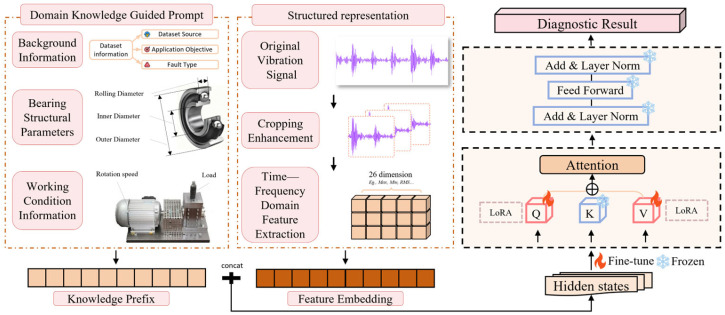
Overall architecture of the KG-SR-LLM framework.

The proposed architecture is based on an LLM and realizes efficient bearing fault diagnosis by integrating three main components: a structured data representation module, a domain knowledge-guided LoRA-Prompt fine-tuning module, and a fault diagnosis module. Figure 1 shows the overall framework of KG-SR-LLM.

### 3.2. Structured Data Representation Module

**Figure 2:**
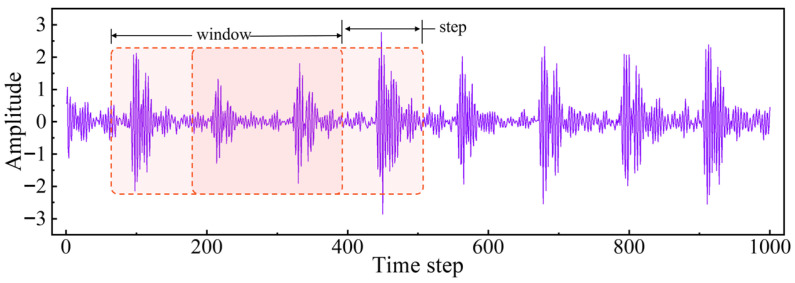
Illustration of cropping-based data augmentation strategy.

Raw vibration data is composed of continuous sequences of acceleration values taken at certain time points or time intervals. A single raw vibration sample generally has tens of thousands or even hundreds of thousands of data points, leading to huge data volumes with considerable redundancy. Single loading of these data will result in heavy memory usage and computational costs, thus decreasing training efficiency. Moreover, as fault samples are rare, normal and faulty samples tend to be seriously imbalanced, which is not conducive to model performance. In consideration of these factors, data augmentation is used to increase the number and diversity of samples. Since the vibration signals exhibit excellent periodicity, this paper follows a cropping-based data augmentation strategy, which divides one-dimensional vibration sequences into fixed-length windows. The enhancing process is shown in Figure 2.

Specifically, this paper sets up multiple window length and step size combinations. Upon the premise that each window covers at least one complete vibration cycle, two window lengths (1024 and 2048) and four step sizes (128, 512, 1024, and 2048) are chosen. These combinations aim to increase sample diversity while ensuring data balance in different datasets. The combination strategy is not fixed; rather it is flexibly regulated towards maximizing the data distribution balance in datasets to achieve better training stability. Owing to the different lengths of the original datasets, even employing adaptive windowing methods, the final numbers of augmented samples remain slightly different among datasets. The specific configurations of windows and steps, and the corresponding numbers of samples of each dataset, are listed in Table 1.

Subsequently, feature extraction is applied to the vibration data. Due to the fact that vibration analysis generally requires specialized engineering knowledge, time–frequency domain features extraction based on predefined formulas allows domain knowledge to be incorporated and enhances the interpretability of features, thus facilitating more effective fault diagnosis. In this work, 14 time-domain features and 12 frequency-domain features are extracted from the vibration signals based on predefined definitions. Time-domain features describe temporal changes of the signals, while frequency-domain features illustrate the distribution of frequencies. Specific feature definitions used in this work are listed in Table 2.

Following the extraction of time–frequency domain features from the vibration data, in this paper these features are further structured into tag–value pairs with semantic attributes, in order to best leverage LLMs for structured information modeling. This transformation converts abstract numerical features into interpretable descriptors with explicit semantic annotations, allowing each feature to be defined in terms of a contextually rich linguistic meaning. Moreover, by using natural language as a unified representational medium, both the features that were extracted and the domain knowledge thereafter are embedded into a shared semantic space, creating a continuous and contextually coherent linguistic representation. This is done to better enable the semantic relations within features to be captured by the model, hence raising the interpretability and expressiveness, as well as diagnostic significance, of the learned representations.

Specifically, in this paper, the 26-dimensional features are expressed in the form of natural language label–value pairs, as illustrated by the following semantic representation example:

The characteristic values of the vibration signal of this bearing are as follows: vibration maximum value: ***2.4470***, vibration minimum value: ***−2.7859***, vibration peak-to-peak value: ***5.2328***, …

This semantic text is included as part of the model input, together with a carefully designed prompt, to create an end-to-end natural language sequence. This application further increases the expressiveness and generalization capability of the LLMs in handling intricate fault patterns.

### 3.3. Domain Knowledge-Guided LoRA-Prompt Fine-Tuning Module

Effective integration of domain expertise and the capabilities of LLMs is still an important task in bearing fault diagnosis. In the diagnosis field, both structural characteristics of bearings and operating conditions play an important role in fault patterns. In addition, distributions of vibration signals are quite different under operating conditions, making it difficult for the model to accurately capture the intricate relationships between domain knowledge and signal representations.

In order to overcome these problems, this paper advances a LoRA-Prompt fine-tuning component guided by domain knowledge. This component explicitly integrates bearings’ structural parameters and operating condition information into the LLM using a combination of LoRA-Prompt fine-tuning and domain-informed prompt design. By integrating domain-specific knowledge into the input and adaptation process of the model, the framework can achieve a more precise fault-relevant feature extraction and greatly improved diagnostic accuracy, as well as a cross-domain generalization capability.

#### 3.3.1. LoRA Fine-Tuning

LoRA [22] is an efficient fine-tuning technique particularly well-suited for adapting large-scale pretrained models. It enables parameter-efficient optimization by introducing low-rank matrices without modifying the core weights of the LLM. The core idea of LoRA is to apply low-rank decomposition on the weight matrices within the LLM, thereby significantly reducing the number of trainable parameters during fine-tuning and lowering both computational and memory overheads. Specifically, LoRA decomposes the weight matrix *W* of the LLM into two low-rank matrices *A* and *B*, and the modified weight is formally defined as

(1) $$W=W_{0}+A\cdot B,A\in {\mathbb{R}}^{d\times r},B\in {\mathbb{R}}^{r\times k}$$

where *W_0_* is the initial weight matrix of the LLM, while *A* and *B* are the trainable low-rank matrices, with r representing the rank used in the decomposition. $A\in {\mathbb{R}}^{d\times r}$ and $B\in {\mathbb{R}}^{r\times k}$, where *d* and *k* correspond to the dimensionality of the weight matrix. LoRA enables efficient fine-tuning by updating only these smaller matrices, thereby preserving the strong representation capability of the original LLM. In this study, the rank r is set to 8, and the scaling factor α is set to 16, achieving a balance between model capacity and parameter efficiency. To reduce the computational overhead and unnecessary model perturbations, LoRA modules are selectively injected into two linear layers within the attention mechanism: the query projection (*q_proj_*) and value projection (*v_proj_*). During fine-tuning, only the parameters introduced by LoRA are updated, while all other parameters remain frozen.

#### 3.3.2. Domain Knowledge-Guided Prompt Design

To strengthen the model’s capability to connect bearing fault features with specific operating conditions, this paper proposes a domain knowledge-guided prompt design strategy. In this approach, the prompt acts as a structured textual input appended to the model’s input, embedding essential prior knowledge to help the model interpret the complex semantics in bearing fault diagnosis. For effective identification of different fault types, the prompt must encapsulate critical information, such as bearings’ structural parameters and contextual details about the operating conditions that affect fault behavior. In this work, the constructed prompt comprises the following key components:

**1. Dataset background information**: This component provides the dataset name, an overview of its source, the number of fault samples, and the fault types included in it. Such background information helps the model in creating an enhanced clarity regarding the goals of classification and the corresponding bearing structures and operating conditions, and hence improves its diagnostic ability.

**2. Structured representation of bearings’ structural parameters**: This component encodes detailed bearing structural parameters, as listed in Table 3. These parameters reflect the geometrical structure and load-bearing characteristics of the bearing, providing essential contextual cues for the model to better understand fault patterns. In addition to standard bearing specifications, this paper also incorporates fault characteristic frequencies, which are known to be critical indicators in vibration-based diagnosis. In general, four characteristic frequencies are considered [23]: Ball Pass Frequency of the Outer race (BPFO), Ball Pass Frequency of the Inner race (BPFI), Ball Spin Frequency (BSF), and Fundamental Train Frequency (FTF) (also referred to as cage speed). These frequencies are computed using the following formulas:

(2) $$f_{BPFO}=\frac{nf_{r}}{2}\left[1-\frac{d}{D}\cos\alpha \right]$$

(3) $$f_{BPFI}=\frac{nf_{r}}{2}\left[1+\frac{d}{D}\cos\alpha \right]$$

(4) $$f_{BSF}=\frac{D}{2d}\left[1-{\left(\frac{d}{D}\cos\alpha \right)}^{2}\right]$$

(5) $$f_{FTF}=\frac{f_{r}}{2}\left[1-\frac{d}{D}\cos\alpha \right]$$

where *f_r_* is the rotational speed, *D* is the pitch diameter of the bearing, *d* is the rolling element diameter, *n* is the number of rolling elements, and *α* is the contact angle between the rolling elements and the races.

**3. Structured representation of operating condition parameters**: This component encodes the bearing’s operating condition parameters, with details outlined in Table 4. These parameters capture the dynamic and environmental conditions influencing bearing behavior, including factors such as load, rotational speed, and temperature. By incorporating this information, the model gains a clearer understanding of real operational contexts, which enhances the reliability and adaptability of its fault diagnosis capabilities.

**Figure 3:**
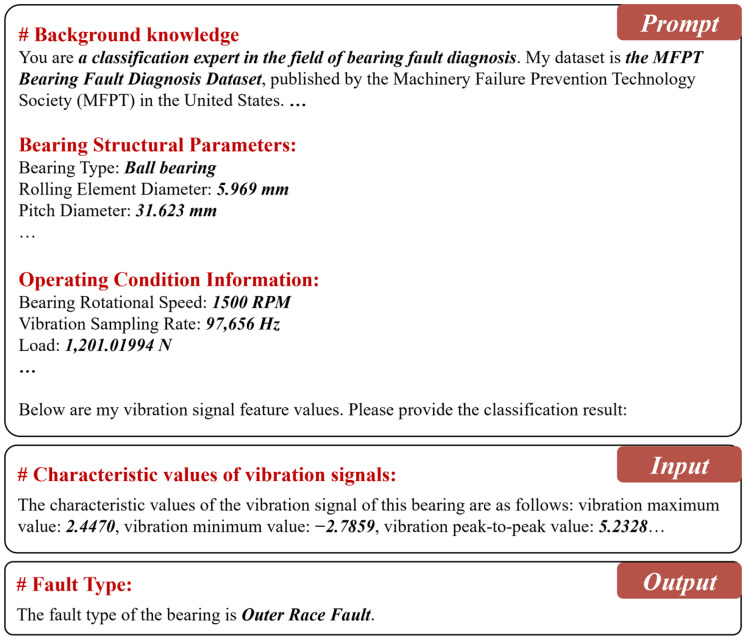
Example of domain prompt construction, input format, and model output.

**4. Prompt Construction**: A complete prompt is constructed by integrating the dataset background, bearing structural parameters, and operating condition information. This combined natural language sequence is then tokenized and transformed into prompt vectors, which are prepended to the input data to provide the LLM with task-specific guidance. By embedding domain knowledge prior to model inference, the prompt helps frame the diagnostic context and steers the model’s attention toward fault-relevant patterns. The complete prompt structure is illustrated in Figure 3.

This approach enables the prompt to directly embed domain-specific knowledge, helping the model build a deeper and more holistic understanding of the bearing’s operational context and fault behavior. Consequently, it improves diagnostic accuracy and significantly enhances the model’s generalization capability across varying operating conditions.

### 3.4. Fault Diagnosis Module

The fault diagnostic module is the core component in KG-SR-LLM. It utilizes the extracted high-quality features, the incorporated domain knowledge, and the fine-tuned LLM for bearing fault diagnosis. The core aim is to determine whether there is a fault present, and if so, to classify it into a particular type.

With targeted fine-tuning for the LLM, the model is capable of reliably identifying an extensive range of fault types. Furthermore, to ensure consistency in the evaluation, the output format is standardized during the course of the fine-tuning for compatibility in multiple datasets and diagnostic scenarios.

#### 3.4.1. Fault Diagnosis Task Definition

The fault diagnosis problem can be viewed as a multi-class classification task, where each class corresponds to a specific fault type or health condition. Let $C=\{C_{1},\;C_{2},\;\ldots ,\;C_{k}\}$ represent the set of all possible categories, with *k* being the total number of classes. Given a processed input sample *x*, the model outputs a probability distribution over the entire category set, which is defined as follows:

(6) $$p\left(y|x\right)=Softmax\left(x\right)$$

where *x* denotes the input data, and *p(y|x)* represents the predicted probability that *x* belongs to category *Ci*. The final fault diagnosis result is obtained by selecting the category with the highest predicted probability:

(7) $$y_{pred}=\arg\underset{i}{\max}p(y_{i}|x)$$

#### 3.4.2. Fault Diagnosis Process

**Figure 4:**
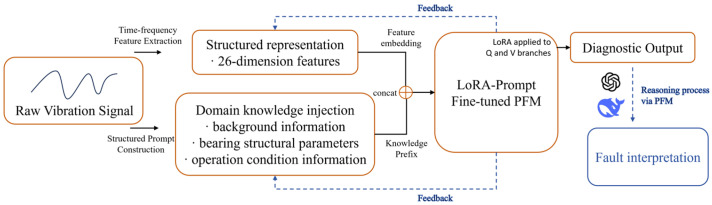
Structured prompt construction and LoRA-LLM-based diagnostic framework with future extensibility.

The fault diagnosis method proposed in this paper is driven by structured prompt construction and a fine-tuned LLM. As illustrated in Figure 4, the framework is designed with a modular and automated architecture, enabling accurate fault diagnosis and future-oriented enhancements. In the diagram, components in orange denote the modules that have been implemented, while those marked with blue dashed outlines represent planned features that are conceptually defined for future development. The full diagnostic workflow consists of the following key steps:

**Step 1: Structured Prompt Construction**: The raw vibration signals are processed in the first place through the module to extract the features in the time–frequency domain, where the statistical features like RMS, kurtosis, and skewness are calculated, producing a 26-dimensional structured feature vector. In parallel, domain-specific knowledge such as bearings’ structural parameters and operating condition information, as well as contextual background knowledge, is fused to create an informed prefix. The above elements are then combined together in order to create the semantic prompt, which is fed into the model.

**Step 2: Fault classification**: The constructed prompt is input into a LoRA-Prompt-enhanced LLM, which interprets the semantic information and performs a classification to evaluate the bearing’s status. If a fault is identified, the model further categorizes it into specific fault types—such as inner race fault, outer race fault, or rolling fault—demonstrating advanced contextual understanding and diagnostic ability.

**Step 3: Output Generation**: Based on the classification, the model provides the bearing’s health status along with the predicted fault type. These interpretable diagnostic results support informed decisions for equipment maintenance and enable proactive measures for early fault prevention.

**Future Perspective**: The existing workflow has attained end-to-end automation—from raw vibration signal processing all the way down to semantic-level diagnostic output—and several smart extension directions for the future development are envisioned in this paper. One direction is the incorporation of a feedback-based refinement mechanism for the prompt. Through the analysis of incorrect predictions or low-confidence predictions, it would be possible for the system to adjust the construction of the prompt autonomously, through reweighting specific features or updating domain knowledge, towards increasingly improving accuracy and flexibility. Another direction for improvement consists of extending the output layer beyond simple fault classification. With the incorporation of reasoning mechanisms, the model would be able to output diagnostic pathways. Furthermore, using the predicted fault type in combination with contextual information, the system would be further enhanced in order to return actionable maintenance advice, such as prioritized inspection, component replacement, or degradation trend forecasting. Overall, these improvements would elevate the framework from intelligent fault diagnosis into more extensive, smart decision support.

## 4. Experiments and Results

### 4.1. Datasets

In this experiment, 11 publicly available bearing fault datasets are employed, covering a wide range of application domains such as industrial, aerospace, and energy systems. These datasets include vibration signals collected under different operating conditions, fault types, and equipment setups, enabling a comprehensive evaluation of the model’s performance and cross-domain generalization capability. The 12th dataset, GS, is used for the cross-dataset experiment. Detailed information on each dataset is presented in Table 5.

### 4.2. Experimental Setup and LLM Choice

The experiments were carried out on a high-performance computing platform equipped with 8 NVIDIA A40 GPUs (Huawei Technologies Co., Ltd., Shenzhen, China), each with 46,068 MB of memory. The software environment included CUDA version 12.6, PyTorch 2.6.0, and Visual Studio Code (VSCode, v1.104.0) as the primary development interface.

In the experiment, this paper adopts Qwen2.5-0.5B-Instruct, an open-source language model developed by Alibaba Academy (Hangzhou, China). With a compact architecture of 0.5 billion parameters, it provides an efficient solution for downstream fine-tuning tasks while maintaining strong capabilities in comprehension of instructions.

### 4.3. Evaluation Indicators

For the complete evaluation of the performance of KG-SR-LLM, four widely accepted evaluation criteria are used: Accuracy, Precision, Recall, and F1-Score. These metrics give an all-around appraisal of the diagnostic ability of the method in terms of overall correctness, accuracy in identifying the faults, sensitivity towards the actual fault cases, and the balance between the precision and recall.

Accuracy, being the basic measure for classification problems, is the proportion of correctly classified samples out of the total number of samples. It is calculated as follows:

(8) $$Accuracy=\;\frac{TP+TN}{TP+TN+FP+FN}$$

where *TP* refers to the number of samples that are actually positive and correctly predicted as positive, *TN* is the number of samples that are actually negative and correctly predicted as negative, *FP* is the number of samples that are actually negative but incorrectly predicted as positive, and *FN* is the number of samples that are actually positive but incorrectly predicted as negative.

Precision measures the proportion of samples predicted as positive by the model that are actually positive. It is calculated as follows:

(9) $$Precision=\;\frac{TP}{TP+FP}$$

Recall represents the proportion of actual positive samples that are correctly predicted as positive by the model. It is calculated as follows:

(10) $$Recall=\;\frac{TP}{TP+FN}$$

F1-Score is the harmonic mean of Precision and Recall and is used to provide a balanced evaluation of the model’s classification performance, especially when dealing with imbalanced datasets. It is calculated as follows:

(11) $$F1-score=2\cdot \frac{Precision\cdot Recall}{Precision+\;Recall}$$

By employing these evaluation metrics, this study systematically evaluates the proposed model’s effectiveness and robustness in the context of bearing fault diagnosis.

### 4.4. Experimental Comparison and Discussion

#### 4.4.1. Fine-Tuning on Multiple Datasets

In order to ensure the validity of KG-SR-LLM, wide-scale tests are performed on 11 public fault datasets. The datasets cover equipment of varying types, fault modes, and operational conditions, providing a foundation for an extensive performance evaluation. Detailed information for all the included datasets is presented in Section 4.1.

In the experiment, all the datasets are divided, with 30% being used for training and 70% for testing. And 10% of the training set is further held out as a validation set. The choice of such a configuration is made based on the fact that the extracted features in the time–frequency domain are very representative, and the model can learn useful diagnostic patterns from relatively few training samples. And the larger test proportion lets the model be comprehensively evaluated on its generalizability for a wide variety of fault types and operating scenarios. Specifically, all the training parts for all 11 datasets are combined into one unified training set, and separately, each dataset is tested for diagnostic performance. In order to avoid data leakage, all the training and testing samples in each experiment are strictly divided. As illustrated in Table 6, the proposed approach has an average diagnostic accuracy as high as 98.36% for all the datasets, in most cases higher than 98%. In addition, we checked the inference efficiency of the proposed method. The average latency for LLM inference is around 0.1162 (seconds/sample), which proves that it is feasible for the proposed method to run in real-time edge computing scenarios. Moreover, we also monitored the resource usage during deployment. The 0.5B-parameter model has a GPU memory footprint of around 1142 MB excluding the KV cache, indicating its lighter weight and its applicability to industrial IoT systems with limited resources.

**Figure 5:**
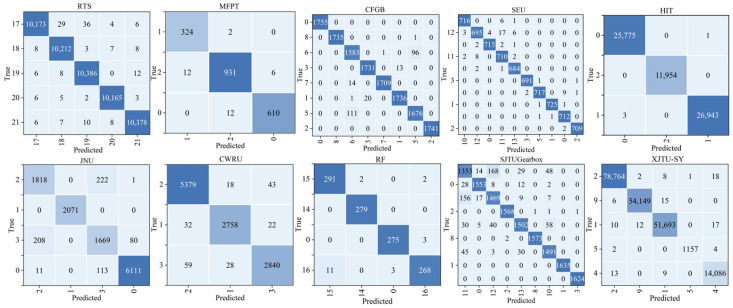
Confusion matrix of KG-SR-LLM across 11 test datasets. The diagonal elements are highlighted in blue, with deeper shades indicating higher numbers of correctly classified samples, in order to better illustrate the classification performance.

To further evaluate the model’s ability to distinguish between different fault types, a confusion matrix is constructed based on the prediction results from all the test samples, as shown in Figure 5. Each cell within the matrix indicates the number of instances associated with a pair of true and predicted labels. Higher values in the off-diagonal entries indicate a greater likelihood of the model misclassifying one fault type as another. Overall, the matrix exhibits a strong diagonal dominance, indicating a high classification accuracy and reliable performance across most fault types. For clarity, note that the confusion matrix includes the combined results of the SEU-Bearing and SEU-Gear datasets, which are grouped together under the label “SEU” due to the difficulty of arranging all 11 datasets in a single matrix. Additionally, Table 7 provides the mapping between the label IDs in the confusion matrix and their corresponding fault types, covering 22 distinct health conditions or fault types.

To verify the advantages and effectiveness of KG-SR-LLM, four classical temporal models—Long Short-Term Memory (LSTM) [33], Transformer [34], CNN, and Residual Network (ResNet) [35]—are used as baseline methods for comparison. These models are all trained and evaluated on the same 11 bearing fault datasets, using identical feature extraction procedures and 26-dimensional statistical feature vectors as input.

For the purposes of an objective and uniform comparison, all the models have the same training setup. Cross-entropy loss is the loss function, and Adam is the optimizer with a learning rate of 0.001. For the purposes of avoiding the problem of overfitting, all the models use a dropout layer. Specifically, LSTM adopts a bidirectional structure with two stacked layers, each containing 256 hidden units. Transformer consists of six encoder layers, with each layer comprising eight attention heads. CNN includes three convolutional blocks, each followed by batch normalization and max pooling, and ends with a fully connected layer for classification. ResNet is constructed with two residual blocks and includes batch normalization and shortcut connections.

In addition, because KG-SR-LLM integrates prior knowledge about datasets and fault types through the prompt, a label masking mechanism is implemented across all baseline models. This ensures that each model only considers fault labels relevant to the current input, thereby aligning the level of prior information and maintaining consistency in the evaluation.

Through training and testing on 11 datasets, this paper compares the performance of KG-SR-LLM with classical baselines, using the same four standard classical evaluation metrics. As the overall trends of these metrics are consistent across models, this paper reports only the accuracy results for clarity and conciseness. The comparison results are presented in Table 8.

The experimental results show that KG-SR-LLM achieves an average accuracy of 98.36% across 11 publicly available bearing fault datasets, clearly outperforming all baseline models—Transformer (89.06%), LSTM (83.67%), CNN (86.34%), and ResNet (75.19%). KG-SR-LLM consistently delivers top performance across all datasets, including those characterized by high noise levels or non-stationary conditions. In comparison, the baseline models exhibit significant variability in accuracy across different datasets, indicating limited robustness. In contrast, KG-SR-LLM maintains a highly stable and reliable performance. These results demonstrate that when LLMs are enhanced with structured feature representations and a domain-knowledge prompt design, they achieve superior diagnostic accuracy and robust cross-domain generalization. This makes KG-SR-LLM particularly suitable for real-world industrial environments involving complex, multi-source vibration data.

#### 4.4.2. Cross-Condition Transfer Experiments

In practical industrial settings, equipment operates under a wide range of complex and fluctuating conditions, with variables such as load and rotational speed changing over time or across different equipment. Therefore, a model’s capacity to generalize to unseen operational scenarios is critical for real-world deployment. To evaluate how well KG-SR-LLM performs under such conditions, cross-condition transfer experiments are conducted using the CWRU dataset. In these experiments, vibration signals collected under different operating conditions are used separately for training and testing, specifically designed to assess the model’s adaptability in the face of shifting operational environments.

**Figure 6:**
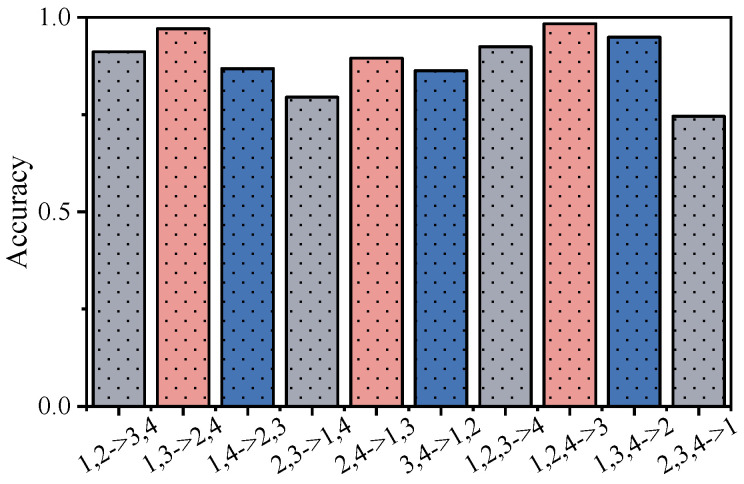
Performance histogram of KG-SR-LLM in cross-condition transfer (CWRU dataset).

Table 9 lists the four distinct operating conditions included in the CWRU dataset, from which 10 different cross-condition transfer combinations are created. In each experiment, two or three conditions serve as source domains for training, while the remaining condition(s) are designated as target domains for testing. To maintain a strict zero-shot setting, no samples from the target condition are introduced during training. The outcomes of these experiments are presented in Table 10. For a clearer visualization of the model’s performance across these transfer scenarios, a bar chart is provided in Figure 6, with each bar representing the accuracy achieved for a specific source-to-target condition transfer.

As shown in Table 10 and Figure 6, KG-SR-LLM achieves an over 85% accuracy in most cross-condition transfer settings, demonstrating strong generalization across different operational environments. However, the method exhibits decreased performance in certain scenarios: for example, when transferring from conditions 2 and 3 to conditions 1 and 4 (accuracy of 79.49%), and from conditions 2, 3, and 4 to condition 1 (accuracy of 74.53%). This decline is primarily attributed to significant distributional gaps between the source and target domains, where the training conditions fail to sufficiently capture the characteristics of the target scenarios. Specifically, the feature distributions of conditions 2 and 3, which reflect medium load and speed levels, do not adequately represent the extremes of condition 1 (no-load, high-speed) and condition 4 (heavy-load, low-speed). These distribution mismatches reduce the model’s ability to generalize effectively to such extremes. Additionally, condition 1 poses further challenges due to its low signal-to-noise ratio and relatively weak fault signatures, particularly for impact-type faults, making it more difficult for the model to transfer knowledge from heavy-load scenarios to the no-load domain.

**Figure 7:**
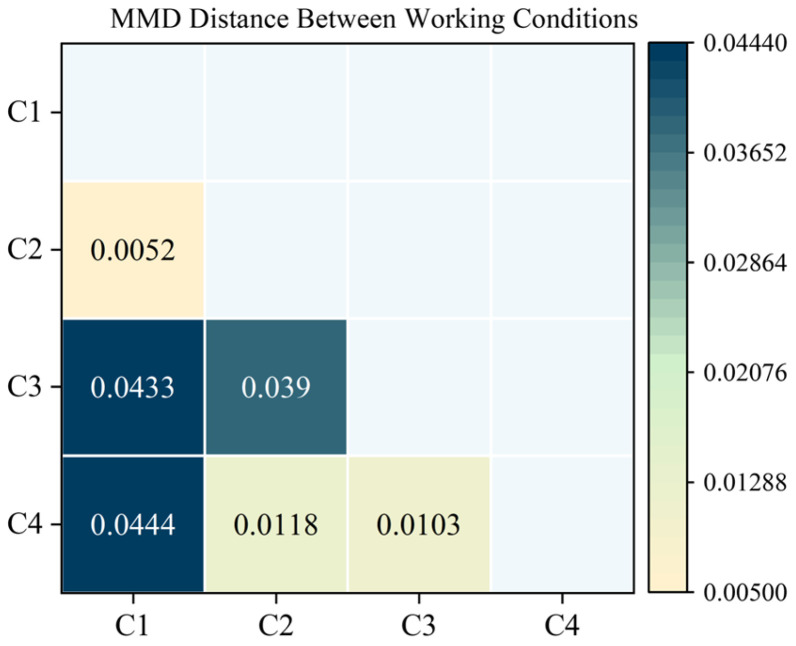
MMD distance matrix between operating conditions (CWRU dataset).

To quantitatively verify the influence of distributional differences on transfer performance, this paper calculates the Maximum Mean Discrepancy (MMD) between different operating conditions and visualizes the results as a distance matrix, as shown in Figure 7. MMD is a non-parametric statistical distance metric based on the kernel method, which measures the divergence between two probability distributions in Reproducing Kernel Hilbert Space (RKHS). Let the source domain samples be $X={\left\{x_{i}\right\}}_{i=1}^{n}$, and the target domain samples be $Y={\left\{y_{j}\right\}}_{j=1}^{m}$, with the kernel function *k*(∙,∙). The kernel function adopted in this paper is the Radial Basis Function (RBF). The unbiased estimation and kernel function of MMD are defined as follows:

(12) $$MMD^{2}\left(X,Y\right)=\frac{1}{n^{2}}\overset{n}{\underset{i=1}{\sum }}\overset{n}{\underset{j=1}{\sum }}k\left(x_{i},x_{j}\right)+\frac{1}{m^{2}}\overset{m}{\underset{i=1}{\sum }}\overset{m}{\underset{j=1}{\sum }}k\left(y_{i},y_{j}\right)+\frac{1}{nm}\overset{n}{\underset{i=1}{\sum }}\overset{m}{\underset{j=1}{\sum }}k\left(x_{i},y_{j}\right)$$

(13) $$k\left(x,y\right)=\exp\left(-\gamma {\left\|x-y\right\|}^{2}\right)$$

where *γ* denotes the kernel width parameter of the RBF kernel. As shown in Figure 7, the distributional distance between operating condition 1 and the other conditions is the most pronounced. This aligns with the performance trends observed in Table 10, further emphasizing that distributional differences significantly impact transfer effectiveness. Although the MMD analysis reflects a substantial shift between condition 1 and the other domains, future studies could focus on narrowing this gap by incorporating adaptive weighting strategies or advanced domain adaptation methods to enhance the model’s generalization capability under unseen operating conditions.

We compare the proposed KG-SR-LLM with popular traditional transfer learning techniques, such as Deep Domain Confusion (DDC) [36] and Domain-Adversarial Neural Networks (DANN) [37], to demonstrate the cross-condition fault diagnosis performance. As presented in Table 11, the accuracy of KG-SR-LLM is slightly lower than that of the baseline transfer learning methods, which is validated by the experimental results. However, one remarkable difference is that our experiments are set to be zero-shot transfer, which does not use any target domain samples during training time, so the test samples in the target domain are never seen by the model before, while methods such as DDC and DANN adopt the unsupervised domain adaptation paradigm, in which they are allowed access to the unlabeled target domain data for the purpose of feature alignment. Our method outperforms these two methods under this more difficult setting, which confirms the effectiveness of our method. Furthermore, in sharp contrast to the conventional transfer learning methods, which can only handle one source–target pair, with the requirement to train a separate learned model, KG-SR-LLM can learn a unified model without the need of re-training the model for every source–target pair, which provides better generalization and practicality for real industrial applications.

#### 4.4.3. Few-Shot Transfer Experiments Across Datasets

**Figure 8:**
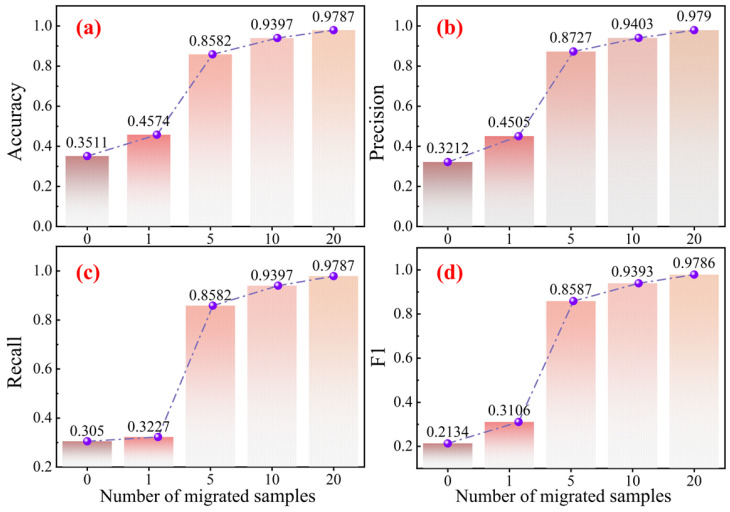
Few-shot transfer performance curves on the GS dataset. Subfigure (**a**) shows the trend of accuracy with different shots, (**b**) shows the trend of precision with different shots, (**c**) shows the trend of recall with different shots, and (**d**) shows the trend of F1 with different shots.

In actual industrial settings, it is both costly and time-consuming to acquire fully labeled fault data, emphasizing the demand for evaluating the performance of a model in data-constrained settings. In order to analyze KG-SR-LLM in data-limited conditions, few-shot transfer experiments are conducted, with the source datasets being the first 11 datasets in Table 5, and the target dataset being the GS dataset (the last dataset in Table 5). These experiments evaluate how the model would perform when trained only using few labeled samples. In these experiments, the number of training samples is set to 0, 1, 5, 10, and 20, respectively. The results are summarized in Table 12, and the corresponding accuracy trends are illustrated through line graphs in Figure 8 to aid visual analysis.

The experimental findings indicate that the model’s performance steadily improves as the number of training samples increases. Notably, under the 10-shot setting, the model reaches an accuracy of 93.97%, showcasing its strong capacity to rapidly adapt to new tasks even with limited supervision. These results affirm the few-shot transfer learning ability of KG-SR-LLM, demonstrating its ability to recognize the key characteristics of the target domain when there is limited data.

**Figure 9:**
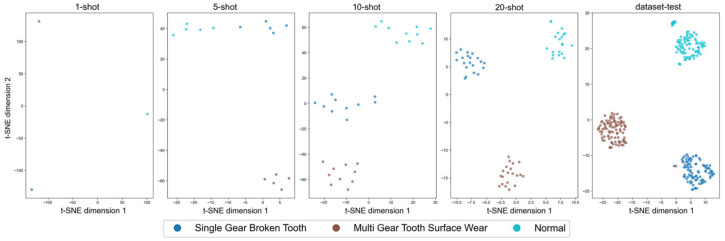
T-SNE feature visualization under varying few-shot settings.

To further evaluate the model’s performance on the differentiation of features in few-shot cases, t-SNE is used for the visualization of the distributions in 1-shot, 5-shot, 10-shot, and 20-shot training examples and the test data, as shown in Figure 9. The visualizations reveal that the groups of features become better differentiated, with clearer fault-type boundaries, with an increase in the number of training samples. This reflects that the model learns increasingly discriminative and stable semantic representations with larger training data quantities. These visualized observations are consistent with the rising classification accuracy, reinforcing the conclusion that the model’s feature extraction and representation capabilities improve as more data becomes available.

**Figure 10:**
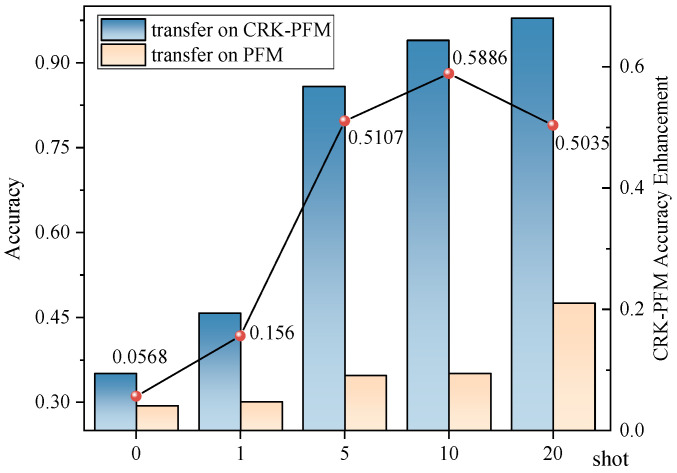
Few-shot transfer results comparison of the unfine-tuned LLM and KG-SR-LLM. The blue bars denote the transfer accuracy of KG-SR-LLM, and the yellow bars denote the transfer accuracy of the unfine-tuned LLM. The line represents the accuracy enhancement achieved by KG-SR-LLM over the unfine-tuned LLM under different few-shot settings.

To further verify the effectiveness of KG-SR-LLM in transfer learning, this paper introduces the unfine-tuned LLM as a baseline for comparison. Experiments are conducted under the same few-shot settings with 0, 1, 5, 10, and 20 training samples, and the results are reported in Table 13. To more intuitively illustrate the performance gap, the classification results are also presented as line graphs in Figure 10. The results show that the unfine-tuned LLM performs significantly worse than the fine-tuned KG-SR-LLM using the proposed method. This demonstrates that the proposed knowledge-guided fine-tuning strategy notably enhances the model’s generalization ability in transfer learning tasks.

### 4.5. Ablation Experiments

Ablation experiments are further carried out to evaluate the significance of each core component in the KG-SR-LLM through their independent removal. The model performance is measured in the absence of each component in order to determine its relative impact on diagnostic accuracy. This method provides a quantitative analysis of the distinct contribution and effectiveness of each module within the overall framework.

#### 4.5.1. Ablation Study of Data Structured Representation Module

In this section, the data structured representation module is removed, and the model is directly fed with the raw vibration sequences as input. The performance of this setting is then compared with the full KG-SR-LLM to evaluate the contribution of the structured feature representation. The same as in previous sections, only the accuracy metric is reported for clarity. The experimental results are presented in Table 14.

As shown in Table 14, the model performs much more poorly when it is fed raw sequences of the vibration instead of the structured time–frequency domain feature text. Average accuracy decreases from 98.36% to 78.99%, and in those with more complex distributions, it decreases by over 30%. The findings underscore the pivotal role played by the structured representation module. It not only strengthens the model’s capability in the extraction of meaningful features in the vibrations but also enhances the semantic interpretability of the input through its transformation into the form of natural language. Such a transformation greatly enhances the model in terms of interpretation and generalizability for different and complicated patterns in the signals.

#### 4.5.2. Ablation Study of Domain Knowledge-Guided LoRA-Prompt Fine-Tuning Module

In this section, the domain knowledge is removed from the prompt design and replaced with generic command prompts, in order to evaluate the impact of domain knowledge’s incorporation on model performance. The results are compared with the full KG-SR-LLM, and the experimental accuracy is presented in Table 15.

As depicted in Table 15, excluding explicit domain knowledge from the input degrades the model accuracy from 98.36% to 97.50%. The reduction is clearer for those tasks highly dependent on operating condition clues. The experiment validates the important contribution made through domain knowledge, as it enables substantial contextual information incorporation, leading the model to better match fault features and varying operational conditions. It indicates that the model is greatly boosted in its generalizability, especially for complex and dynamic diagnostic cases, through the incorporation of domain-specific knowledge.

## 5. Conclusions

This paper offers a novel fault diagnosis framework using LLMs to address limitations of conventional methods in cross-condition and cross-equipment scenarios. With its combination of a structured data presentation and LoRA-Prompt fine-tuning that is guided using domain knowledge, as well as taking advantage of LLMs’ strong capability for learning representations, the method is exceptionally effective in various datasets. Additionally, it shows robust generalization to cross-domain fault diagnosis problems.

Comprehensive experiments with 11 publicly available bearing fault datasets demonstrate the strong generalizability and flexibility of KG-SR-LLM, with the method achieving an average accuracy of 98.36%, far outperforming conventional deep learning baselines. The method performs strongly on cross-condition and cross-dataset transfer scenarios, highlighting its real-world usability in industry. Ablation experiments affirm the effectiveness of the two major components of the framework, which highlight the significance of coupling semantic representation with a domain-specific engineering prompt.

In conclusion, this paper tackles the problem of using LLMs for fault diagnosis and proposes a scalable, highly robust framework that integrates a structured input representation with expert-knowledge fine-tuning. Furthermore, the proposed method is not confined to the application of fault diagnosis but rather can be generally applied to a variety of time-series data analyses. While the method presented in this paper is effective, there are still some limitations. First, the existing method is still based on a few labeled samples and therefore does not completely achieve zero-shot transfer between datasets. Second, both the possibility of and accuracy for practical industrial applications of the method need to be further validated, given that the existing test platform could hardly reproduce all of the complexity of reality. Third, valuable sources of information, which are also available in practice (e.g., maintenance logs, technical datasheets, or operational records), have not been incorporated by the framework yet and could greatly enrich the diagnostic process. Fourth, the method has not considered multimodal data representations (vibration + image or textual information, etc.) that facilitate diagnostic and cross-domain generalization. We will also work to address these limitations in our follow-up work.

## Figures and Tables

**Table 1:** Window and step configurations for data augmentation across datasets.

Dataset	Window	Step	Samples Number
MFPT	1024	512	2710
CFGB	2048	1024	19,890
HIT	1024	512	94,068
JNU	1024	512	17,577
XJTU-SY	2048	1024	285,665
SEU-Bearing	2048	2048	5110
SEU-Gear	2048	2048	5110
XJTUGearbox	2048	1024	20,638
CWRU	2048	2048	16,022
RTS	2048	2048	73,554
GS	1024	128	582
RF	1024	128	1620

**Table 2:** Definitions of extracted time-domain and frequency-domain features.

**Time-Domain Features**	**Mathematical Formulas**
Maximum value	$M_{max}=\max\left\{x_{1},x_{2},\ldots ,x_{i}\right\}$
Minimum value	$M_{min}=\min\left\{x_{1},x_{2},\ldots ,x_{i}\right\}$
Peak-to-peak value	$M=M_{max}-M_{min}$
Mean value	$M_{mean}=\frac{1}{N}\overset{N}{\underset{n=1}{\sum }}x\left(n\right)$
Standard deviation	$M_{std}=\sqrt{\frac{1}{N-1}\overset{N}{\underset{n=i}{\sum }}{\left[x\left(n\right)-M_{mean}\right]}^{2}}$
Root amplitude	$M_{ra}={\left(\frac{1}{N}\overset{N}{\underset{n=1}{\sum }}\sqrt{\left|x\left(n\right)\right|}\right)}^{2}$
Root mean square (RMS)	$M_{rms}=\sqrt{\frac{1}{N}\sum_{n=1}^{N}x^{2}(n)}$
Absolute mean value	$M_{aavg}=\frac{1}{N}\overset{N}{\underset{n=1}{\sum }}\left|x\left(n\right)\right|$
Skewness	Mske=1N−1∑n=1N(xn−Mmean)3Mstd 3
Kurtosis	Mkur=1N−1∑n=1N(xn−Mmean)4Mstd 4
Crest factor	$M_{peak}=max\left|x\left(n\right)\right|/M_{rms}$
Wave factor	$M_{\mathrm{wave}}=M_{rms}/M_{aavg}$
Impulse factor	$M_{\mathrm{pulse}}=max\left|x\left(n\right)\right|/M_{aavg}$
Margin factor	$M_{margin}=max\left|x\left(n\right)\right|/M_{ra}$
**Frequency-Domain Features**	**Mathematical Formulas**
Mean frequency	$p_{mean}=\frac{1}{N}\overset{N}{\underset{n=1}{\sum }}s\left(n\right)$
Frequency variance	$p_{\mathrm{var}}=\frac{1}{N-1}\overset{N}{\underset{n=1}{\sum }}{\left[s\left(n\right)-p_{mean}\right]}^{2}$
Frequency skewness	$p_{\mathrm{ske}}=\frac{1}{Np_{\mathrm{var}}^{3/2}}\overset{N}{\underset{n=1}{\sum }}{\left[s\left(n\right)-p_{\mathrm{mean}}\right]}^{3}$
Frequency kurtosis	$p_{\mathrm{kur}}=\frac{1}{Np_{\mathrm{var}}^{2}}\overset{N}{\underset{n=1}{\sum }}{\left[s\left(n\right)-p_{\mathrm{mean}}\right]}^{4}$
Spectral centroid	$p_{\mathrm{bar}}=\overset{N}{\underset{n=1}{\sum }}f_{i}s\left(n\right)/\overset{N}{\underset{n=1}{\sum }}s\left(n\right)$
Frequency standard deviation	$p_{\mathrm{std}}=\sqrt{\overset{N}{\underset{n=1}{\sum }}{\left(f_{i}-p_{\mathrm{bar}}\right)}^{2}s\left(n\right)/N}$
Root mean square frequency	$p_{\mathrm{rms}}=\sqrt{\overset{N}{\underset{n=1}{\sum }}f_{i}^{2}s\left(n\right)/\overset{N}{\underset{n=1}{\sum }}s\left(n\right)}$
Average frequency	$p_{avg}=\sqrt{\overset{N}{\underset{n=1}{\sum }}f_{i}^{4}s\left(n\right)/\overset{N}{\underset{n=1}{\sum }}f_{i}^{2}s\left(n\right)}$
Spectral smoothness	$p_{\mathrm{reg}}=\overset{N}{\underset{n=1}{\sum }}f_{i}^{2}s\left(n\right)/\sqrt{\overset{N}{\underset{n=1}{\sum }}s\left(n\right)/\overset{N}{\underset{n=1}{\sum }}f_{i}^{4}s\left(n\right)}$
Spectral variation	$p_{\mathrm{vari}}=p_{\mathrm{std}}/p_{\mathrm{bar}}$
8th-order spectral moment	$p_{\mathrm{eight}}=\overset{N}{\underset{n=1}{\sum }}{\left(f_{i}-p_{bar}\right)}^{3}s\left(n\right)/Np_{std}^{3}$
16th-order spectral moment	$p_{sixteen}=\overset{N}{\underset{n=1}{\sum }}{\left(f_{i}-p_{bar}\right)}^{4}s\left(n\right)/Np_{std}^{4}$

**Table 3:** Structural and geometric parameters of bearings.

Argument	Unit
Bearing Model	\
Bearing Type	\
Rolling Diameter	mm
Pitch Diameter	mm
Rolling Elements	piece
Contact Angle	degree
Inner Diameter	mm
Outer Diameter	mm
Mean Diameter	mm
Dynamic Load	N
Static Load	N
BPFO	Hz
BPFI	Hz
BSF	Hz
FTF	Hz

**Table 4:** Experimental operating condition parameters of bearings.

Argument	Unit
Rotation Speed	RPM
Vibration Sampling Rate	Hz
Current Sampling Rate	Hz
Mechanical Sampling Rate	Hz
Temperature Sampling Rate	Hz
Load	N
Radial Force	N

**Table 5:** Overview of bearing fault diagnosis datasets.

Dataset	Equipment Type	Sampling Rate	Fault Type Number
MFPT [24,25]	Industrial Bearing	48.8/97.6 kHz	3
CFGB	Gearbox Bearing	50 kHz	8
HIT [26]	Aerospace Bearing	25 kHz	3
JNU [27]	Motor Bearing	50 kHz	4
XJTU-SY [28]	Industrial Bearing	25.6 kHz	5
SEU-Bearing [29]	Gearbox Bearing	5120 Hz	5
SEU-Gear [29]	Gearbox Bearing	5120 Hz	5
XJTUGearbox [30]	Gearbox Bearing	20.48 kHz	9
CWRU [31]	Motor Bearing	12/48 kHz	4
RTS	Drive shafts	4096 Hz	5
RF [32]	Rotor System	2048 Hz	4
GS	Gearbox Bearing	10 kHz	3

**Table 6:** Diagnostic performance of KG-SR-LLM across 11 public datasets.

Dataset	Accuracy	Precision	Recall	F1
MFPT	98.31%	98.32%	98.31%	98.31%
CFGB	98.36%	98.15%	98.15%	98.15%
HIT	99.99%	99.99%	99.99%	99.99%
JNU	94.84%	94.79%	94.84%	94.81%
XJTUGearbox	96.72%	95.31%	95.30%	95.30%
XJTU-SY	99.93%	99.94%	99.94%	99.94%
SEU-Bearing	99.36%	99.36%	99.36%	99.36%
SEU-Gear	98.41%	98.42%	98.41%	98.41%
CWRU	98.19%	98.20%	98.19%	98.19%
RTS	99.66%	99.66%	99.66%	99.66%
RF	98.15%	98.18%	98.15%	98.15%
Average	98.36%	98.21%	98.21%	98.21%

**Table 7:** Mapping between fault-type labels and descriptions.

ID	Fault Type
0	Normal
1	Inner Race Fault
2	Outer Race Fault
3	Rolling Fault
4	Cage Fault
5	Inner and Outer Race Composite Fault
6	Inner Race and Rolling Composite Fault
7	Outer Race and Rolling Composite Fault
8	Inner and Outer Race and Rolling Composite Fault
9	Inner and Outer Race and Rolling and Cage Composite Fault
10	Broken Tooth
11	Tooth Surface Wear
12	Tooth Root Crack
13	Missing Gear
14	Imbalance
15	Contact Friction
16	Misalignment
17	No Imbalance
18	Slight Imbalance
19	Moderate Imbalance
20	Severe Imbalance
21	Pronounced Imbalance

**Table 8:** Accuracy comparison of KG-SR-LLM and baseline models on 11 datasets. The best results in each row are highlighted in bold.

Dataset	KG-SR-LLM	TRANSFORMER	LSTM	CNN	ResNet
MFPT	**98.31%**	96.05%	93.04%	92.25%	72.64%
CFGB	**98.36%**	98.22%	98.06%	98.20%	70.24%
HIT	**99.99%**	76.65%	66.02%	63.99%	67.59%
JNU	**94.84%**	91.02%	88.15%	87.52%	87.95%
XJTUGearbox	**96.72%**	91.18%	88.13%	88.57%	85.03%
XJTU-SY	**99.93%**	99.23%	97.70%	96.54%	94.62%
SEU-Bearing	**99.36%**	90.72%	82.61%	86.05%	68.58%
SEU-Gear	**98.41%**	90.72%	82.61%	86.05%	68.58%
CWRU	**98.19%**	92.23%	79.45%	83.22%	82.48%
RTS	**99.66%**	87.53%	79.52%	83.91%	69.63%
RF	**98.15%**	66.14%	65.08%	83.42%	59.70%
Average	**98.36%**	89.06%	83.67%	86.34%	75.19%

**Table 9:** Description of CWRU operating conditions.

Working Condition	Speed/RPM	Load/HP
C1	1797	0
C2	1772	1
C3	1750	2
C4	1730	3

**Table 10:** Cross-condition transfer results on the CWRU dataset.

Source Condition	Target Condition	Accuracy	Precision	Recall	F1
1, 2	3, 4	91.16%	91.19%	91.16%	91.14%
1, 3	2, 4	97.05%	97.12%	97.05%	97.04%
1, 4	2, 3	86.87%	86.79%	86.87%	86.70%
2, 3	1, 4	79.49%	79.37%	79.49%	79.37%
2, 4	1, 3	89.48%	89.56%	89.48%	89.40%
3, 4	1, 2	86.32%	86.30%	86.32%	86.13%
1, 2, 3	4	92.41%	93.01%	92.41%	92.45%
1, 2, 4	3	98.31%	98.33%	98.31%	98.31%
1, 3, 4	2	94.91%	94.92%	94.91%	94.88%
2, 3, 4	1	74.53%	74.90%	74.53%	74.66%

**Table 11:** Accuracy comparison of KG-SR-LLM and baseline transfer learning models. The best results in each row are highlighted in bold.

Source Condition	Target Condition	KG-SR-LLM	DDC	DANN
1, 2	3, 4	**91.16%**	85.57%	90.09%
1, 3	2, 4	**97.05%**	93.64%	94.62%
1, 4	2, 3	86.87%	91.58%	**93.07%**
2, 3	1, 4	79.49%	89.30%	**89.95%**
2, 4	1, 3	89.48%	91.18%	**93.18%**
3, 4	1, 2	86.32%	88.33%	**91.58%**
1, 2, 3	4	92.41%	90.89%	**93.16%**
1, 2, 4	3	**98.31%**	90.59%	95.10%
1, 3, 4	2	94.91%	**95.68%**	94.94%
2, 3, 4	1	74.53%	**84.65%**	84.38%

**Table 12:** Few-shot transfer results under different shot settings (GS dataset). The best results in each column are highlighted in bold.

Shot	Accuracy	Precision	Recall	F1
0	35.11%	32.12%	30.50%	21.34%
1	45.74%	45.05%	32.27%	31.06%
5	85.82%	87.27%	85.82%	85.87%
10	93.97%	94.03%	93.97%	93.93%
20	**97.87%**	**97.90%**	**97.87%**	**97.86%**

**Table 13:** Performance of unfine-tuned LLM under few-shot transfer (GS dataset). The best results in each column are highlighted in bold.

Shot	Accuracy	Precision	Recall	F1
0	29.43%	22.31%	26.24%	20.03%
1	30.14%	20.79%	30.14%	23.36%
5	34.75%	36.80%	34.75%	30.62%
10	35.11%	35.16%	35.11%	33.25%
20	**47.52%**	**45.78%**	**47.52%**	**44.17%**

**Table 14:** Performance comparison between KG-SR-LLM and original vibration data. The best results in each row are highlighted in bold.

Dataset	KG-SR-LLM	Original Vibration Data
MFPT	**98.31%**	98.16%
CFGB	**98.36%**	53.57%
HIT	**99.99%**	61.48%
JNU	**94.84%**	99.15%
XJTUGearbox	**96.72%**	46.44%
XJTU-SY	**99.93%**	99.84%
SEU-Bearing	**99.36%**	65.17%
SEU-Gear	**98.41%**	61.84%
CWRU	**98.19%**	95.20%
RTS	**99.66%**	98.91%
RF	**98.15%**	89.15%
Average	**98.36%**	78.99%

**Table 15:** Performance comparison between KG-SR-LLM and without knowledge. The best results in each row are highlighted in bold.

Dataset	KG-SR-LLM	Without Knowledge
MFPT	**98.31%**	97.26%
CFGB	**98.36%**	98.23%
HIT	**99.99%**	99.95%
JNU	**94.84%**	90.65%
XJTUGearbox	**96.72%**	96.33%
XJTU-SY	**99.93%**	99.90%
SEU-Bearing	**99.36%**	97.90%
SEU-Gear	**98.41%**	98.10%
CWRU	**98.19%**	98.00%
RTS	**99.66%**	99.48%
RF	**98.15%**	96.74%
Average	**98.36%**	97.50%

## Data Availability

The data presented in this study are available on request from the corresponding authors.
